# A community-based survey of *Toxoplasma gondii* infection among pregnant women in rural areas of Taiz governorate, Yemen: the risk of waterborne transmission

**DOI:** 10.1186/s40249-017-0243-0

**Published:** 2017-02-13

**Authors:** Mohammed A. K. Mahdy, Lina M. Q. Alareqi, Rashad Abdul-Ghani, Samira M. A. Al-Eryani, Abdullah A. Al-Mikhlafy, Abdulsalam M. Al-Mekhlafi, Fawzya Alkarshy, Rohela Mahmud

**Affiliations:** 1 Tropical Disease Research Center, University of Science and Technology, Sana’a, Yemen; 2 Department of Parasitology, Faculty of Medicine and Health Sciences, Sana’a University, Sana’a, Yemen; 3 Department of Parasitology, Faculty of Medicine, University of Malaya, 50603 Kuala Lumpur, Malaysia; 4 Department of Community Medicine, Faculty of Medicine, University of Science and Technology, Sana’a, Yemen; 5 Yemeni-Swedish Hospital, Ministry of Public Health and Population, Taiz, Yemen

**Keywords:** *Toxoplasma gondii*, Rural communities, Pregnant women, Waterborne transmission, Yemen

## Abstract

**Background:**

*Toxoplasma gondii* is a zoonotic coccidian parasite causing morbidity and mortality. In Yemen, *T. gondii* infection has been reported among pregnant women seeking healthcare in the main cities. However, no data are available on the prevalence of *T. gondii* infection and its associated risk factors among pregnant women in the rural communities of the country. Thus, the present study aimed to determine the seroprevalence of *T. gondii* and identify its risk factors among pregnant women in the rural communities of Taiz governorate, Yemen.

**Methods:**

A total of 359 pregnant women living in the rural communities of Taiz governorate were enrolled in this study by house-to-house visits. Data were collected using a pre-designed questionnaire, and blood samples were collected and tested for the detection of anti- *T. gondii* IgM and IgG antibodies by enzyme-linked immunosorbent assay.

**Results:**

The prevalence of *T. gondii* infection among pregnant women in this study was 46.2% (166/359). Bivariate analysis identified the age of  ≥ 30 years (odds ratio [OR] = 1.7; 95% confidence interval [CI] = 1.09–2.65, *P* = 0.019) and unimproved water sources (OR = 2.2; 95% CI = 1.10–4.55, *P* = 0.023) as factors associated with *T. gondii* infection among pregnant women. The multivariable analysis, however, identified unimproved water sources as an independent risk factor (adjusted OR = 2.4; 95% CI = 1.16–5.0, *P* = 0.018) associated with *T. gondii* infection among pregnant women.

**Conclusions:**

Pregnant women in the rural communities of Taiz, Yemen are at high risk of contracting *T. gondii* infection. Unimproved water sources (wells, water streams and water tanks) are significantly associated with *T. gondii* infection and should be considered in prevention and control strategies, especially among pregnant women.

**Electronic supplementary material:**

The online version of this article (doi:10.1186/s40249-017-0243-0) contains supplementary material, which is available to authorized users.

## Multilingual abstracts

Please see Additional file 1 for translations of the abstract into the five official working languages of the United Nations.

## Background

*Toxoplasma gondii* is a zoonotic coccidian parasite that is distributed worldwide, with about a third of the human population having a chronic infection [1, 2]. Humans are infected by ingesting oocysts directly or in contaminated water or food, or by eating undercooked meat containing tissue cysts [3, 4]. Blood transfusion and organ transplantation are also potential routes of transmission [5]. *T. gondii* infection is asymptomatic in healthy adults but can cause severe complications in immunocompromised individuals [6]. Moreover, primary infection in pregnant women may lead to congenital toxoplasmosis, with the highest incidence rates being reported in low-income African countries and parts of the Middle East [7]. Congenital toxoplasmosis following primary infection during pregnancy is the most important aspect of human toxoplasmosis, possibly leading to abortion, stillbirth, congenital anomalies as well as neurological and ocular complications [1]. *T. gondii* seroprevalence among pregnant women or females of reproductive age is high in different parts of the world. In their review on the global status of *T. gondii* infection among pregnant women, Pappas et al. [8] cited *T. gondii* seroprevalence rates of 6.1–77.5% among women who were pregnant or of reproductive age in the Americas, 8.2–63.2% in Europe, 25.3–75.2% in Africa and 0.8–> 60% in Asia and Oceania.

Diagnosis of *T. gondii* infection is commonly established by serological testing for immunoglobulin M (IgM) and immunoglobulin G (IgG) antibodies [2]. IgM antibodies are produced in the first week after infection and decline to undetectable levels within months. Although its diagnostic value in determining acute infection is limited as it may persist for years [9, 10], IgM negativity rules out acute infection. IgG antibodies appear 2–3 weeks after IgM antibodies, and their levels then decrease, resulting in lifelong persistence of low residual titers [11]. IgG avidity was introduced to overcome false-positive IgM reactions and the inability of IgG to discriminate between past and recent infections, where low-avidity IgG antibodies usually indicate a recent infection [12, 13]. However, because low-avidity IgG antibodies may persist for long periods of time, this hampers their utility to diagnose acute infection [14, 15]. For more correct diagnosis of *T. gondii* infection, a clinical situation-based diagnostic strategy has been suggested. However, the detection of IgG and IgM antibodies is still the most commonly used approach for primary screening [16, 17]. This could be further improved by the development of diagnostic kits based on recombinant multi-epitope antigens [17] or on immunoreactive proteins [18, 19].

In Yemen, the large population of stray cats represents a significant source of environmental contamination because of the oocysts they shed in their faeces. Oocyst-contaminated water sources put the entire human population at risk of infection [3]. Nevertheless, no data are available on the prevalence of *T. gondii* infection and its associated risk factors in the rural communities of Yemen, especially among pregnant women, with previous reports focusing primarily on hospital-based cases in urban communities [20, 21]. Thus, this study aimed to investigate the prevalence and risk factors for *T. gondii* infection among pregnant women living in the rural communities of Taiz, Yemen.

## Methods

### Study area

**Fig. 1:**
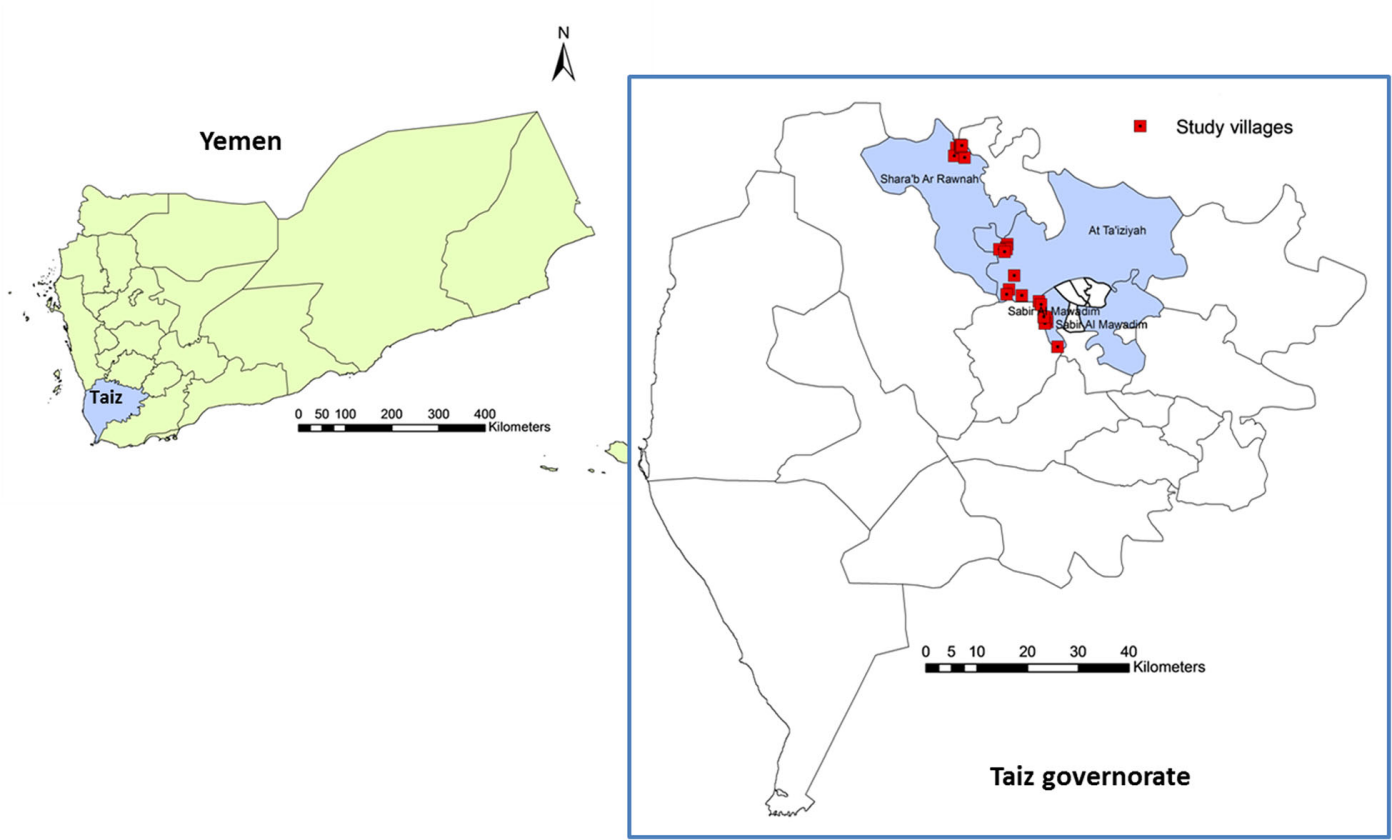
Map of Yemen showing the location of Taiz governorate and the study area

A cross-sectional, community-based survey was conducted in 56 easily accessible rural areas of Taiz, Yemen, in the period from May 2012 to February 2014. Taiz is the most populous city in Yemen located at the geographical coordinates of 13°34′44″ N 44°01′19″ E. It is situated at an elevation of about 1 400 metres above the Red Sea level, southwest of Sana’a (see Fig. 1).

All pregnant women in the surveyed communities were invited to participate in this study by house-to-house visits. The coordinates of pregnant women’s houses were determined using Global Positioning System software (GPSMAP® 60CSx, Garmin, Tonopah, AZ, USA).

### Data and blood collection

A pre-designed questionnaire was used to collect data about the participating women’s age, gestational age, education level, employment, type of water source that their household uses and whether they reared animals. They were also asked about their habit of chewing khat, the leaves of the shrub *Catha edulis*, which are chewed like tobacco.

Water sources were classified into improved water sources (piped water connected to the household) and unimproved water sources (wells, water streams and water tanks), following the criteria of UNICEF/WHO Joint Monitoring Programme for Water Supply and Sanitation [22]. Data were also collected about the participants’ history of miscarriage.

Blood specimens were collected in pre-labelled tubes, and sera were separated after clotting by centrifugation at 3 000 rounds per minute for 10 min. Sera were then preserved at -20 °C until the performance of serological investigations.

### Serological investigations

Sera were tested for anti- *T. gondii* IgM and IgG antibodies using commercial enzyme-linked immunosorbent assay (ELISA) kits (NovaTec Immundiagnostica GmbH, Dietzenbach, Germany), as according to the manufacturer’s instructions. Antibody titers were determined following the normal range established by the manufacturer, and presented as positive or negative. Positive and negative controls were included in each of the ELISA tests to ensure the integrity of the reagents and technical performance.

### Statistical analysis

Data were entered and analysed using the IBM SSPS Statistics for Windows, version 22.0 (IBM Corp., Armonk, NY, USA). Descriptive data obtained from the questionnaires and laboratory investigations were presented as frequencies and percentages. Differences and associations between categorical variables were tested using the Pearson’s chi-square test. Unadjusted odds ratios (ORs) and 95% confidence intervals (CIs) were reported in the bivariate analysis. Variables with *P*-values < 0.08 were entered into a forward logistic regression model to calculate the adjusted ORs and identify the independent predictors of infection. *P*-values < 0.05 were considered statistically significant.

## Results

### Characteristics of the study population

Of the pregnant women enrolled in the present study, 31.5%, 34.9% and 33.5% were in the first, second and third trimesters, respectively. The median age was 26 years (interquartile range: 22–30). More than half of the women reported rearing animals and having the habit of chewing khat. History of miscarriage was reported by 45.7% of the women, and the majority of the women’s households had unimproved water sources (see Table 1).

**Table 1:** Bivariate analysis of factors associated with *T. gondii* IgG seropositivity among pregnant women in the rural communities of Taiz, Yemen (2012–2014)

Variable	*N*	*n* (%)	OR (95% CI)	*P*-value
Age (years)				
14–29	241	101 (41.9)	1	
≥ 30	118	65 (55.1)	1.7 (1.09–2.65)	0.019
Education (*n* = 356)				
Primary school or above	67	32 (47.8)	1	
No formal education	289	131 (45.3)	0.9 (0.53–1.54)	0.719
Parity				
≤ 2	109	48 (44.0)	1	
> 2	250	118 (47.2)	1.4 (0.72–1.79)	0.580
Gestational age (*n* = 355)				
First trimester	112	54 (48.2)	1	
Second trimester	124	54 (43.5)	0.8 (0.50–1.38)	0.470
Third trimester	119	55 (46.2)	0.9 (0.55–1.55)	0.760
History of miscarriage (*n* = 356)				
No	193	86 (44.6)	1	0.613
Yes	163	77 (47.2)	1.1 (0.73–1.69)	
Employment (*n* = 356)				
Employed	10	3 (30.0)	1	
Unemployed	346	160 (46.2)	2.0 (0.51–7.89)	0.309
Type of household water source (*n* = 357)				
Improved	41	12 (29.3)	1	
Unimproved	316	152 (48.1)	2.2 (1.10–4.55)	0.023^a^
Storing water at household (*n* = 315)				
No	43	21 (48.8)	1	
Yes	272	121 (44.5)	0.8 (0.44–1.60)	0.594
Rearing animals (*n* = 351)				
No	150	63 (42.0)	1	
Yes	201	96 (47.8)	1.3 (0.82–1.93)	0.283
Chewing khat (*n* = 354)				
No	141	56 (39.7)	1	
Yes	213	105 (49.3)	1.48 (0.96–2.27)	0.076

*N*, number examined, *n*, number of IgG-positive women

^a^Confirmed as an independent risk factor by multivariable analysis

### *T. gondii* seropositivity and associated risk factors among the study population

The seroprevalence of *T. gondii* infection among pregnant women as indicated by IgG antibodies was 46.2% (166/359). However, anti-*T. gondii* IgM antibodies were detected in 12 samples (3.3%): combined with anti- *T. gondii* IgG antibodies in 1.4% (5/359) and alone in 1.9% (7/359) of samples. Bivariate analysis showed a significant association between *T. gondii* infection and the age group of  ≥ 30 years old (OR = 1.7, 95% CI = 1.09–2.65, *P* = 0.019). Pregnant women living in households with unimproved water sources had a twofold higher risk of being infected with *T. gondii* compared with those using improved water sources (OR = 2.2; 95% CI = 1.10–4.55, *P* = 0.023). Khat chewers showed a higher, but statistically non-significant, rate of contracting *T. gondii* infection than non-chewers (OR = 1.48; 95% CI = 0.96–2.27, *P* = 0.076). On the other hand, the participants’ gestational age, parity, previous history of miscarriage, contact with animals, education status and employment status were not significantly associated with *T. gondii* infection. The multivariable analysis identified unimproved water sources as the independent risk factor associated with *T. gondii* infection among pregnant women (adjusted OR = 2.4; 95% CI = 1.16–5.0, *P* = 0.018) (Table 1).

## Discussion

This is the first community-based survey to determine the seroprevalence of *T. gondii* infection and its associated risk factors among pregnant women in the rural areas of Yemen. Past exposure to *T. gondii* infection was found in 46.2% of the surveyed pregnant women in the Taiz governorate, as revealed by the seropositivity of IgG antibodies. IgM seronegativity among the majority of IgG-positive women rules out acute infection. This finding is comparable to that recently reported (44%; 59/134) among pregnant women admitted to an obstetrics clinic in urban Taiz [21]. It remains uncertain whether the very low seropositivity rates of IgM antibodies, either alone or in combination with IgG antibodies, indicate an acute infection among pregnant women in the present study because neither seroconversion nor IgG avidity testing was performed. About a half of the pregnant women were IgG-negative and are, therefore, not immune to infection. Thus, there is a high risk of acquiring primary infection with *T. gondii* during pregnancy, increasing the risk of congenital toxoplasmosis [16].

The present study revealed a significant association between *T. gondii* infection and unimproved water sources in the rural communities of Taiz. The infection was about twofold more likely in pregnant women using unimproved water sources compared with those using improved water sources. This finding is consistent with a report from the rural communities of Guatemala, which revealed a significant association between the high prevalence of *T. gondii* infection and drinking unfiltered well water [23]. Similarly, the association of *T. gondii* infection with drinking unboiled well water was also reported from rural Poland [24], which was thereafter confirmed by the isolation of parasite oocysts from drinking water in the same rural communities [25]. Moreover, water has been suggested as a source of *T. gondii* infection in several other studies conducted in different countries such as Brazil, Turkey, São Tomé and Príncipe and Taiwan [26–29].

The association between unimproved water sources and past exposure to *T. gondii* infection in the present study could be attributed to the large population of stray cats in the rural communities of Yemen. Cats shed millions of oocysts that are resistant to destruction and can survive in the soil for long periods under ordinary environmental conditions [3]. Water sources can be contaminated by runoff from the soil contaminated by excreta of infected cats, leading to widespread dissemination of *T. gondii* infection. Oocyst-mediated waterborne transmission of *T. gondii* has been reported in different parts of the world, including Atlanta, Georgia [30], Panama [31], Canada [32], Brazil [33] and Ethiopia [34].

The significantly higher infection rate of *T. gondii* among pregnant women aged 30 years or above in the present survey is consistent with those reported in previous studies elsewhere [35–38]. This indicates a higher risk of exposure of older women to *T. gondii* infection, which could possibly be due to the fact that older women conduct more activities involving contact with unimproved water sources. A previous study showed that adult women in rural Yemen are the main water bearers (Mahdy et al., unpublished data). This postulation is supported by the significant association between both older age and unimproved water sources with *T. gondii* infection observed in the present study.

The habit of chewing khat was not significantly associated with past exposure to *T. gondii* infection in this study, though a larger proportion of those infected with *T. gondii* were khat chewers. The role of unwashed vegetables as vehicles for transmitting *T. gondii* oocysts is well documented [4, 39, 40]. Khat could also play this transmission role, as it may become contaminated with oocysts from the environment or if it is washed with contaminated water. It is noteworthy that the local customs of cooking meat under high pressure and eating well-cooked meat exclude the possibility of *T. gondii* infection through eating meat in these rural communities. In addition, animal contact does not appear to be a predictor of *T. gondii* infection among pregnant women, as revealed by the absence of a significant association between rearing animals and *T. gondii* infection in the present study.

Parity, gestational age, education status, employment status and previous history of miscarriage were not significantly associated with *T. gondii* infection among pregnant women in this study. However, in a recent study [21], previous miscarriages among women in Taiz were found to be associated with *T. gondii* infection. This could be attributed to the fact that a large proportion of pregnant women in that clinic-based study might have sought medical care after abortion.

## Conclusions

In conclusion, about half of the pregnant women in the rural communities of Taiz governorate surveyed in this study showed a previous exposure to *T. gondii* infection, with unimproved water sources determined as being the significant source of infection. Therefore, intervention strategies for prevention and control should target such sources. In addition, educating pregnant women about the importance of boiling water could be an effective control measure in these rural communities.

## Additional file

Additional file 1: Multilingual abstracts in the five official working languages of the United Nations. (PDF 922 kb)

## Abbreviations

CI: Confidence interval
ELISA: Enzyme-linked immunosorbent assay
IgG: Immunoglobulin G
IgM: Immunoglobulin M
OR: Odds ratio
SPSS: Statistical Packages for Social Sciences
