# Supplementary material for: A community-based survey of Toxoplasma gondii infection among pregnant women in rural areas of Taiz governorate, Yemen: the risk of waterborne transmission
Source: Infect Dis Poverty. 2017 Feb 13;6:26. doi: 10.1186/s40249-017-0243-0 (PMC5304399; doi:10.1186/s40249-017-0243-0)
Supplement: Additional file 1: — Multilingual abstracts in the five official working languages of the United Nations. (PDF 922 kb) [file 40249_2017_243_MOESM1_ESM.pdf]

Translation of the abstract into the five official working languages of the United Nations

استطلاع مجتمعي عن عدوى التوكسوبلازما بين النساء الحوامل في المناطق الريفية في محافظة تعز، اليمن: خطر انتقال العدوى المنقولة عن طريق المياه

محمد عبد ك مهدي، لينا م. س. العريقي، رشاد عبد الغني، سميرة محمد عبد الرحمن الأرياني، عبد الله مخلافي، عبد السلام محمد المخلافي، فوزية القرشي، روهيلا محمود

ملخص

خلفية: التوكسوبلازما هو طفيل كوكسيديا حيواني المصدر يسبب الاعتلال والوفيات. في اليمن، تم الإبلاغ عن إصابات بالتوكسوبلازما بين النساء الحوامل اللائي ذهبن للحصول على الرعاية الصحية في المدن الرئيسية. ومع ذلك، لا تتوفر أية بيانات حول انتشار عدوى التوكسوبلازما وعوامل الخطر المرتبطة بها بين النساء الحوامل في المجتمعات الريفية من البلاد. لذا تهدف الدراسة الحالية إلى تحديد الانتشار المصلي للتوكسوبلازما وتحديد عوامل الخطر بين النساء الحوامل في المجتمعات الريفية في محافظة تعز، اليمن.

الطرق: تم تسجيل ما مجموعه 359 من النساء الحوامل المصابات في المجتمعات الريفية في محافظة تعز في هذه الدراسة من خلال زيارات منزلية. وقد تم جمع البيانات باستخدام استبيان مصمم مسبقا، وجمعت عينات الدم واختبار للكشف عن التوكسوبلازما مفتش باستخدام الأجسام المضادة IgG وIgM للجلوبولين المناعي عن طريق فحص مقايسة المُمْتَزِ المناعي.

النتائج: كان معدل انتشار عدوى التوكسوبلازما بين النساء الحوامل في هذه الدراسة 46.2٪ (359/166). التحليل ذو المتغيرين أظهر أن السن ≤ 30 عاما (نسبة الأرجحية [OR] = 1.7، فاصل الثقة 95٪ [CI] = 1.09-2.65، P = 0.019) ومصادر المياه غير المعالجة (OR = 2.2، 95٪ CI = 1.10- 4.55، P = 0.023) والعوامل المرتبطة بعدوى التوكسوبلازما بين النساء الحوامل. حدد التحليل متعدد المتغيرات مصادر المياه غير المعالجة كعامل مخاطر مستقل (OR معدل = 2.4، 95٪ CI = 1.16 حتى 5.0، P = 0.018) المرتبطة بعدوى التوكسوبلازما بين النساء الحوامل.

الاستنتاجات: النساء الحوامل في المجتمعات الريفية في محافظة تعز، اليمن هن الأكثر عرضة لخطر الإصابة بالتوكسوبلازما. وترتبط مصادر المياه غير المعالجة (الآبار ومجاري المياه وخزانات المياه) إلى حد كبير بالإصابة التوكسوبلازما وينبغي النظر في استراتيجيات الوقاية والمكافحة، وخاصة بين النساء الحوامل.

Translated from English version into Arabic by Mahmoud Sami, through

TRANSLATORS
WITHOUT BORDERS

也门塔伊兹省农村地区孕妇感染刚地弓形虫的社区调查：水传播的风险

Mohammed A. K. Mahdy, Lina M. Q. Alareqi, Rashad Abdul-Ghani, Samira M. A. Al-Eryani, Abdullah Al-Mekhlafi, Abdulsalam M. Almekhlafi, Fawzya Alkarshy, Rohela Mahmud

摘要

引言：刚地弓形虫（*Toxoplasma gondii*）是一种人兽共患的球虫寄生虫，能导致人类发病和死亡。在也门，已有在主要城市寻求卫生保健的孕妇中发现感染刚地弓形虫的报道。然而，在该国农村社区孕妇弓形虫感染的患病率及其相关危险因素方面尚无可用数据。本研究旨在确定也门塔伊兹省农村社区孕妇刚地弓形虫的血清阳性率，并确定其危险因素。

方法：通过逐户拜访，本研究共纳入居住在也门塔伊兹省农村社区的 359 名孕妇。研究人员通

过预先设计的问卷收集相关数据，收集血液标本，采用酶联免疫吸附试验测试刚地弓形虫 IgG 和 IgM。

结果：该地区孕妇刚地弓形虫患病率为 46.2%（166/359）。双变量分析显示，≥30 岁（*OR*=1.7，95% *CI*=1.09–2.65，*P*=0.019），未处理的水源为孕妇感染刚地弓形虫的危险因素（*OR*=2.2, 95% *CI*=1.10–4.55， *P*=0.023）。多变量分析显示，未处理的水源是孕妇感染刚地弓形虫独立危险因素（校正 *OR*=2.4, 95% *CI*=1.16–5.0，*P*=0.018）。

结论：也门塔伊兹省农村社区孕妇感染刚地弓形虫风险较高。未处理的水源（水井、溪流和水箱）与刚地弓形虫感染显著相关，在该病的预防和控制策略中，特别是针对孕妇，应该考虑这一危险因素。

Translated from English version into Chinese by Xin-Yu Feng, edited by Pin Yang, through

CHINESE CENTER FOR DISEASE CONTROL AND PREVENTION
NATIONAL INSTITUTE OF PARASITIC DISEASES

Enquête communautaire de l'infection par le *Toxoplasma gondii* parmi des femmes enceintes dans des régions rurales du gouvernorat de Taiz, Yémen : le risque d'une transmission par l'eau

Mohammed A. K. Mahdy, Lina M. Q. Alareqi, Rashad Abdul-Ghani, Samira M. A. Al-Eryani, Abdullah Al-Mekhlafi, Abdulsalam M. Almekhlafi, Fawzya Alkarshy, Rohela Mahmud

Résumé

Contexte : le *Toxoplasma gondii* est un parasite coccidien zoonotique à l'origine de cas de morbidité et de mortalité. Au Yémen, l'infection par le *T. gondii* a été signalée chez des femmes enceintes en recherche de soins de santé dans les principales villes. Il n'existe néanmoins aucune donnée relative à la prévalence de l'infection par le *T. gondii* et ses facteurs de risque associés parmi des femmes enceintes au sein des communautés rurales du pays. La présente étude s'attache donc à déterminer la séroprévalence du *T. gondii* et à identifier ses facteurs de risque parmi les femmes enceintes au sein des communautés rurales du gouvernorat de Taiz, Yémen.

Méthodes : au total, 359 femmes enceintes vivant au sein de communautés rurales du gouvernorat de Taiz ont été recrutées par cette étude dans le cadre de visites à domicile. Les données ont été collectées à l'aide d'un questionnaire prédéfini et des échantillons de sang ont été prélevés avant d'y déterminer la présence d'anticorps IgG et IgM dirigés contre le *T. gondii* dans le cadre d'un essai d'immuno-absorption enzymatique.

Résultats : la prévalence de l'infection par le *T. gondii* parmi des femmes enceintes dans le cadre de cette étude atteignait 46,2 % (166/359). L'analyse bivariée a permis de déterminer que l'âge de ≥ 30 ans (rapport des cotes [OR] = 1,7, intervalle de confiance [*IC*] à 95 % = 1,09–2,65, *P* = 0,019) et les sources d'eau non améliorées (*OR* = 2,2, *IC* à 95 % = 1,10–4,55, *P* = 0,023) constituaient des facteurs associés à l'infection par le *T. gondii* parmi des femmes enceintes. L'analyse multivariée a permis de déterminer que les sources d'eau non améliorées constituaient un facteur de risque indépendant (*OR* ajusté = 2,4, *IC* à 95 % = 1,16–5,0, *P* = 0,018) associé à l'infection par le *T. gondii* parmi des femmes enceintes.

Conclusions : les femmes enceintes au sein de communautés rurales du gouvernorat de Taiz au Yémen sont exposées à un risque élevé de contraction d'une infection par le *T. gondii*. Les sources d'eau non améliorées (puits, ruisseaux et réservoirs d'eau) sont associées de manière significative à l'infection par le *T. gondii* et il convient d'en tenir compte dans le cadre de stratégies de prévention et de lutte, notamment parmi les femmes enceintes.

Translated from English version into French by eric ragu, through

TRANSLATORS
WITHOUT BORDERS

Общинного обследования по инфекциям *Toxoplasma gondii* среди беременных женщин в сельских районах провинции Taiz governorate, Йемен: риск передачи инфекции через воду

Mohammed A. K. Mahdy, Lina M. Q. Alareqi, Rashad Abdul-Ghani, Samira M. A. Al-Eryani, Abdullah Al-Mekhlafi, Abdulsalam M. Almekhlafi, Fawzya Alkarshy, Rohela Mahmud

Реферат

Фон: *Toxoplasma gondii является* паразитом зоонозных кокцидий, который может вызвать заболеваемости и смертности. В Йемене, инфекции *T. gondii* были сообщены среди беременных женщин, которые обращаются за медицинской помощью в крупных городах. Однако, нет никаких данных о распространенности и ассоциированных факторах риска инфекций *T. gondii* среди беременных женщин в сельских общинах страны. Таким образом, настоящее исследование, направленное на определение серотипа *T. gondii* и выявить факторы риска среди беременных женщин в сельских общинах провинции Taiz, Йемен.

Методы: В данное исследование были включены дома до дома поездки в общей сложности 359 беременных женщин, проживающих в сельских общинах провинции Taiz. Данные были собраны с использованием заранее разработанного вопросника, и образцы крови были собраны и проверены для обнаружения *T. gondii* IgG и IgM-антител с помощью твердофазного иммуноферментного анализа.

Результаты: Распространенность *T. gondii* -инфекции среди беременных женщин в этом исследовании составила 46,2% (166/359). В двумерного анализа определили возраст ≥ 30 лет (отношение шансов [*OR*] = 1.7, 95% доверительный интервал [*CI*] = 1.09–2.65, *P* = 0,019) и неулучшенные источники воды (*OR*= 2,2, 95% *CI* = 1.10–4.55, *P* = 0,023) как факторы, связанные с инфекциями *T. gondii* среди беременных женщин.

Заключение: Беременные женщины в сельских общинах провинции Taiz, Йемен подвергаются высокому риску заражения инфекцией *T. gondii*. Неусовершенствованные источники воды (колодцы, потоки воды и емкости для воды) в значительной степени связаны с инфекциями *T. gondii* и должны рассматриваться в стратегии профилактики и контроля, особенно среди беременных женщин.

Translated from English version into Russian by Hao-Qi Zhang

Estudio sobre infección por *toxoplasma gondii* en mujeres embarazadas de las comunidades rurales de la gobernación de Ta'izz, Yemen: el riesgo de la transmisión por el agua

Mohammed A. K. Mahdy, Lina M. Q. Alareqi, Rashad Abdul-Ghani, Samira M. A. Al-Eryani, Abdullah Al-Mekhlafi, Abdulsalam M. Almekhlafi, Fawzya Alkarshy, Rohela Mahmud

Resumen

Antecedentes*:* El *toxoplasma gondii* es un parásito coccidio causante de una zoonosis que genera morbilidad y mortalidad. En Yemen, se ha informado sobre la presencia de infección por *t. gondii* en mujeres embarazadas que van a atenderse a los centros de salud de las principales ciudades. Sin embargo, no se cuenta con datos sobre la prevalencia de la infección por *t. gondii* y los factores de riesgo asociados a esta en las mujeres embarazadas de las comunidades rurales del país. El presente estudio tiene entonces por finalidad determinar la seroprevalencia de *t. gondii* e identificar los factores de riesgo asociados a este en mujeres embarazadas de las comunidades rurales de la gobernación de Ta'izz, Yemen.

Métodos: Por medio de visitas puerta a puerta, se inscribió a un total de 359 mujeres embarazadas residentes en las comunidades rurales de la gobernación de Ta'izz. Se recabaron datos empleando un cuestionario previamente diseñado, y se reunieron y analizaron muestras de sangre para detectar anticuerpos IgG e IgM de *t. gondii* por medio de un enzimoinmunoensayo de adsorción.

Resultados: La prevalencia de infección por *t. gondii* en mujeres embarazadas en este estudio fue del 46,2% (166/359). En el análisis en función de dos variables se identificaron la edad de $\geq$ 30 años (índice de disparidad [OR] = 1.7, 95% intervalo de confianza [*CI*] = 1.09–2.65, *P* = 0.019) y las fuentes de suministro de agua no tratada (*OR* = 2.2, 95% *CI* = 1.10–4.55, *P* = 0.023) como factores de riesgo asociados a la infección por *t. gondii* en mujeres embarazadas. En el análisis en función de múltiples variables se identificaron las fuentes de suministro de agua no tratada como factor de riesgo independiente (*OR* ajustado = 2.4, 95% *CI* = 1.16–5.0, *P* = 0.018) asociado a la infección por *t. gondii* en mujeres embarazadas.

Conclusiones: Las mujeres embarazadas residentes en las comunidades rurales de la gobernación de Ta'izz, Yemen corren grave riesgo de contraer infección por *t. gondii*. Las fuentes de suministro de agua no tratada (pozos, corrientes de agua y tanques de agua) tienen significativa incidencia en la infección por *t. gondii* y deberán ser tomadas en cuenta al diseñar las estrategias de prevención y combate a la enfermedad, en especial en relación con las mujeres embarazadas.

Translated from English version into Spanish by Mónica Algazi, through

TRANSLATORS
WITHOUT BORDERS
